# Supplementary figures and images for: N-acetylglucosamine supplementation fails to bypass the critical acetylation of glucosamine-6-phosphate required for Toxoplasma gondii replication and invasion
Source: PLoS Pathog. 2024 Jun 20;20(6):e1011979. doi: 10.1371/journal.ppat.1011979 (PMC11218972; doi:10.1371/journal.ppat.1011979)

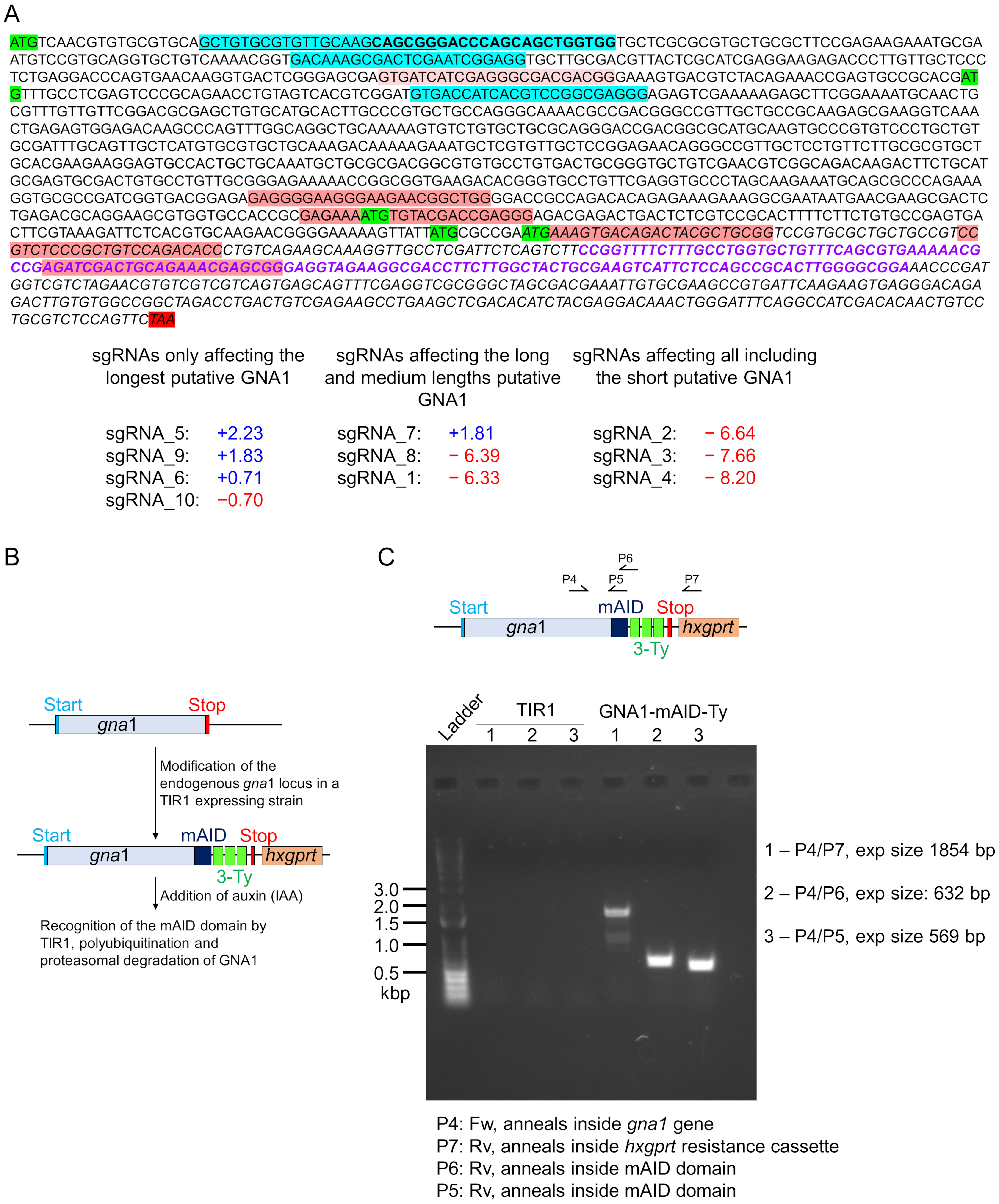

Supplement: S1 Fig — A) GNA1 coding sequence as found on ToxoDB. The initial start codon is highlighted in green, the stop codon in red. Four additional ‘in-frame’ start codons were found and are also highlighted by green shading. Blue (guide with positive score) and red shading (guide with negative score; intensity of shading indicating score) highlights the sequence of single guide RNAs (sgRNA) used in the genome-wide fitness screen. For overlapping guides, the first guide is shown as underlined, the second guide is shown in bold. The name/number of the guides and their respective fitness score is provided. The listing is from left to right and from top to bottom in the order of their appearance in the coding sequence. Note that the first 4 guides only affect the longest putative GNA1 product, while the last 4 guides affect all GNA1 products, including the shortest potential GNA1 protein (highlighted in italic). The acetyltransferase domain, needed for the catalytic activity is highlighted in purple and bold. B) Schematic depiction of the GNA1 locus and its modification through insertion of a mini auxin inducible degron (mAID) domain, a 3-Ty tag and a hxgprt resistance cassette for selection. C) Schematic showing the binding sites of primers used to validate the successful modification of the GNA1 locus and integration PCR, showing the expected bands following amplification with the indicated primers. Sequences of these primers are listed in S1 Table. (TIF) [file ppat.1011979.s001.tif]

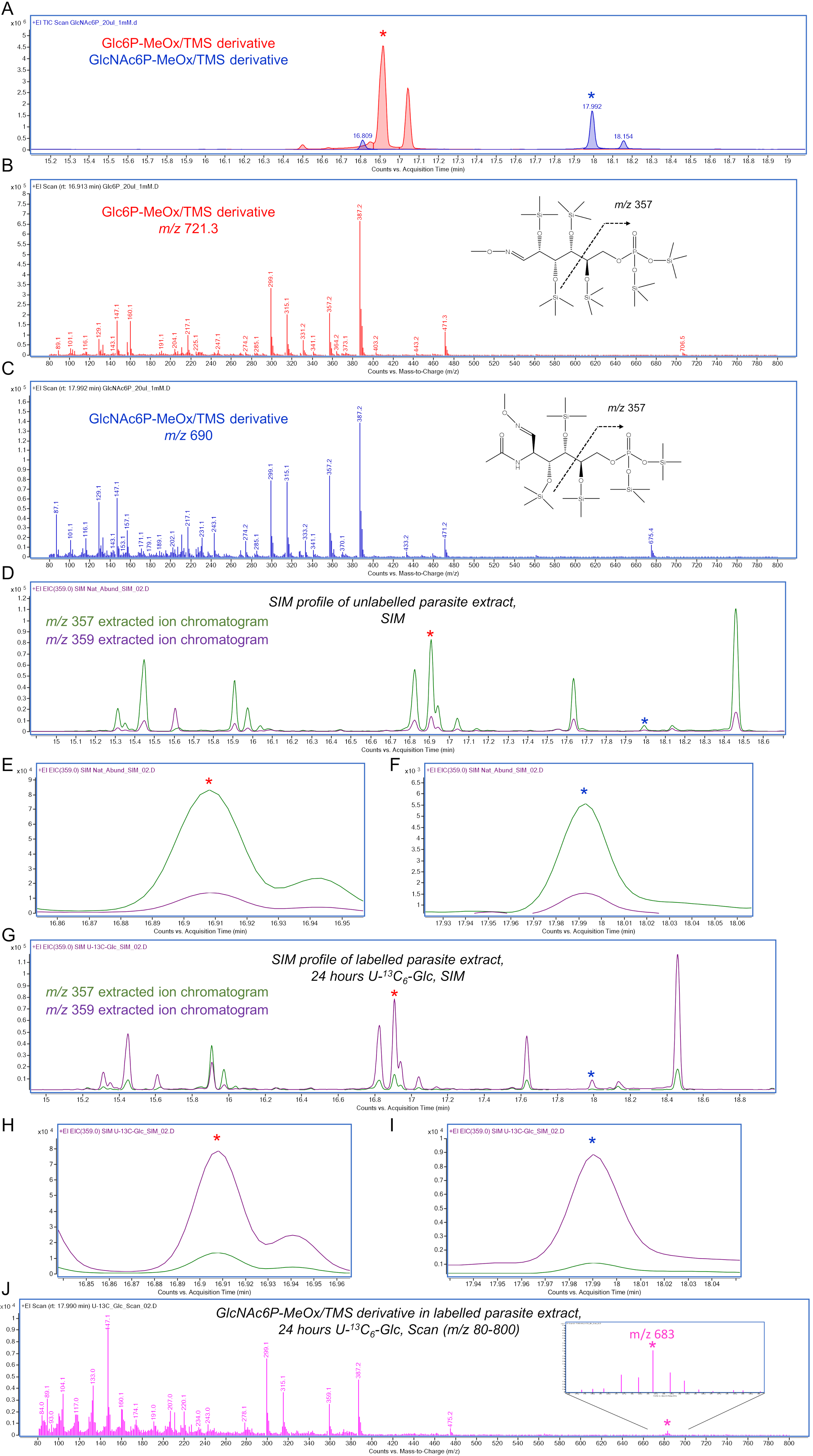

Supplement: S2 Fig — A) Authentic standards of glucose-6-phosphate (Glc6P) and N-acetylglucosamine-6-phosphate (GlcNAc6P) were derivatised via methoximation and silylatation. Their overlayed gas chromatography-mass spectrometry (GC-MS) chromatograms are shown. B-C) Structures of the derivatives and their ion spectra at the indicated retention times (see asterisk in A) are provided. Note that both derivatives share a common fragment of m/z 357, which contains 2 carbons of the sugar but both compounds also exhibit unique fragments, including the ions of m/z 706 and m/z 675, corresponding to the [M-15]+ ions following loss of a methyl group from the Glc6P and GlcNAc6P derivatives, respectively. D) Representative selected ion monitoring (SIM) ion profile, showing traces of the ions m/z 357 and m/z 359 of an unlabelled parasite extract. The peaks corresponding to the Glc6P and GlcNAc6P derivatives are highlighted with an asterisk. E, F) Detail of the ion profiles shown in D for Glc6P and GlcNAc6P, respectively. G) Representative selected ion monitoring (SIM) ion profile, showing the traces of the ions m/z 357 and m/z 359 in an extract of parasites labelled during intracellular growth for 24 hours with 10 mM U-13C6-Glc. The peaks corresponding to the Glc6P and GlcNAc6P derivatives are highlighted with an asterisk. H-I) Detail of the ion profiles shown in G for Glc6P and GlcNAc6P, respectively. J) Representative ion spectrum (Scan, m/z 80–800) for the GlcNAc6P derivative from the same sample as shown in G-I. Note that the ion m/z 675 shifts to m/z 683, indicating that all carbons, including those of the acetyl group are labelled from the provided U-13C6-Glc. (TIF) [file ppat.1011979.s002.tif]

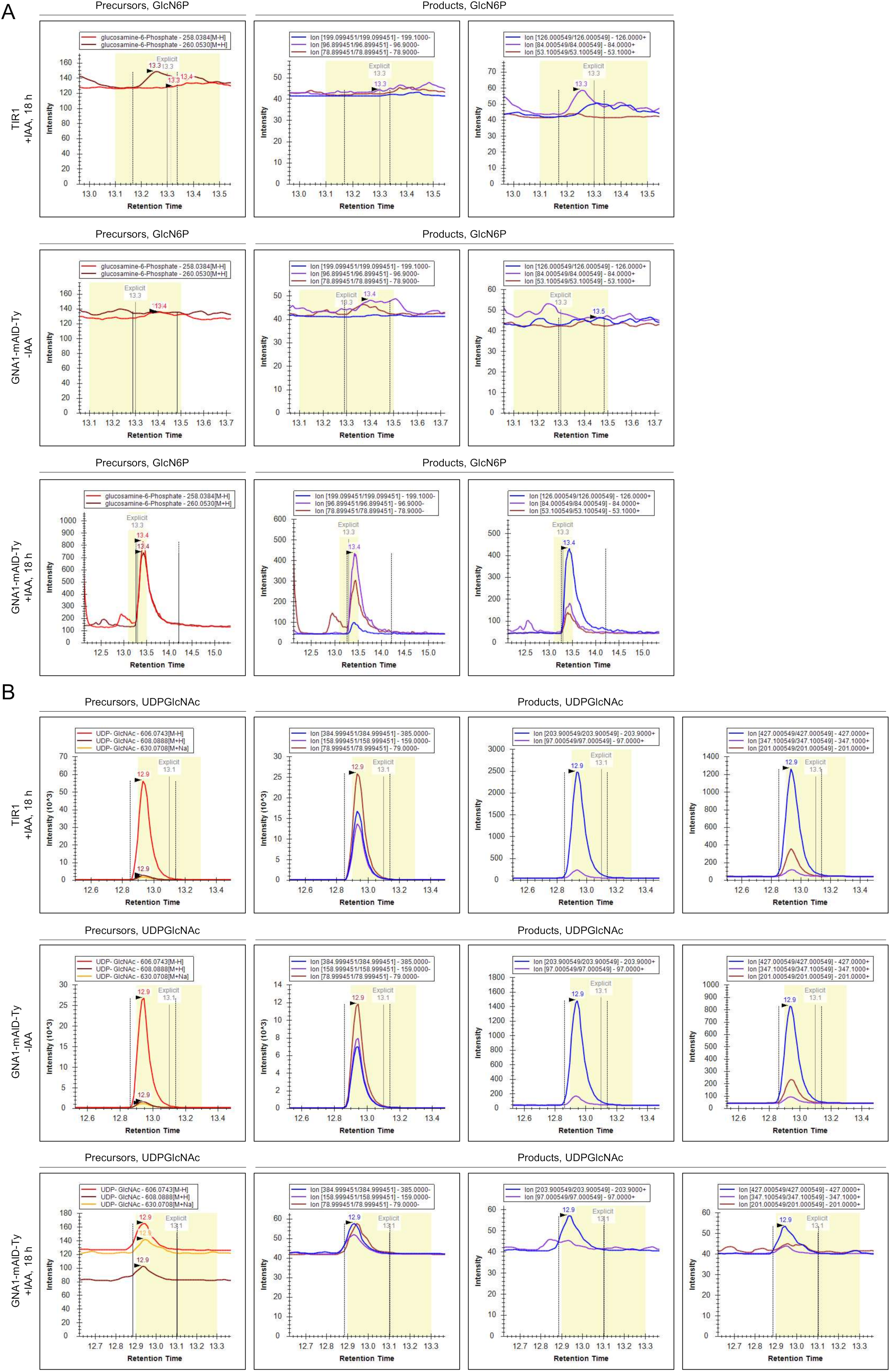

Supplement: S3 Fig — A) Signal obtained for GlcN6P precursor and product ions in parasites depleted in GNA1 for 18 hours (GNA1-mAID-Ty +IAA) and the relevant controls (TIR1 +IAA, GNA1-mAID-Ty–IAA) by LC-MS. B) Signal obtained for UDP-GlcNAc precursor and product ions in parasites depleted in GNA1 for 18 hours (GNA1-mAID-Ty +IAA) and the relevant controls (TIR1 +IAA, GNA1-mAID-Ty–IAA) by LC-MS. The retention times and transitions are listed in the panels and can also be found in S2 Table. (TIF) [file ppat.1011979.s003.tif]

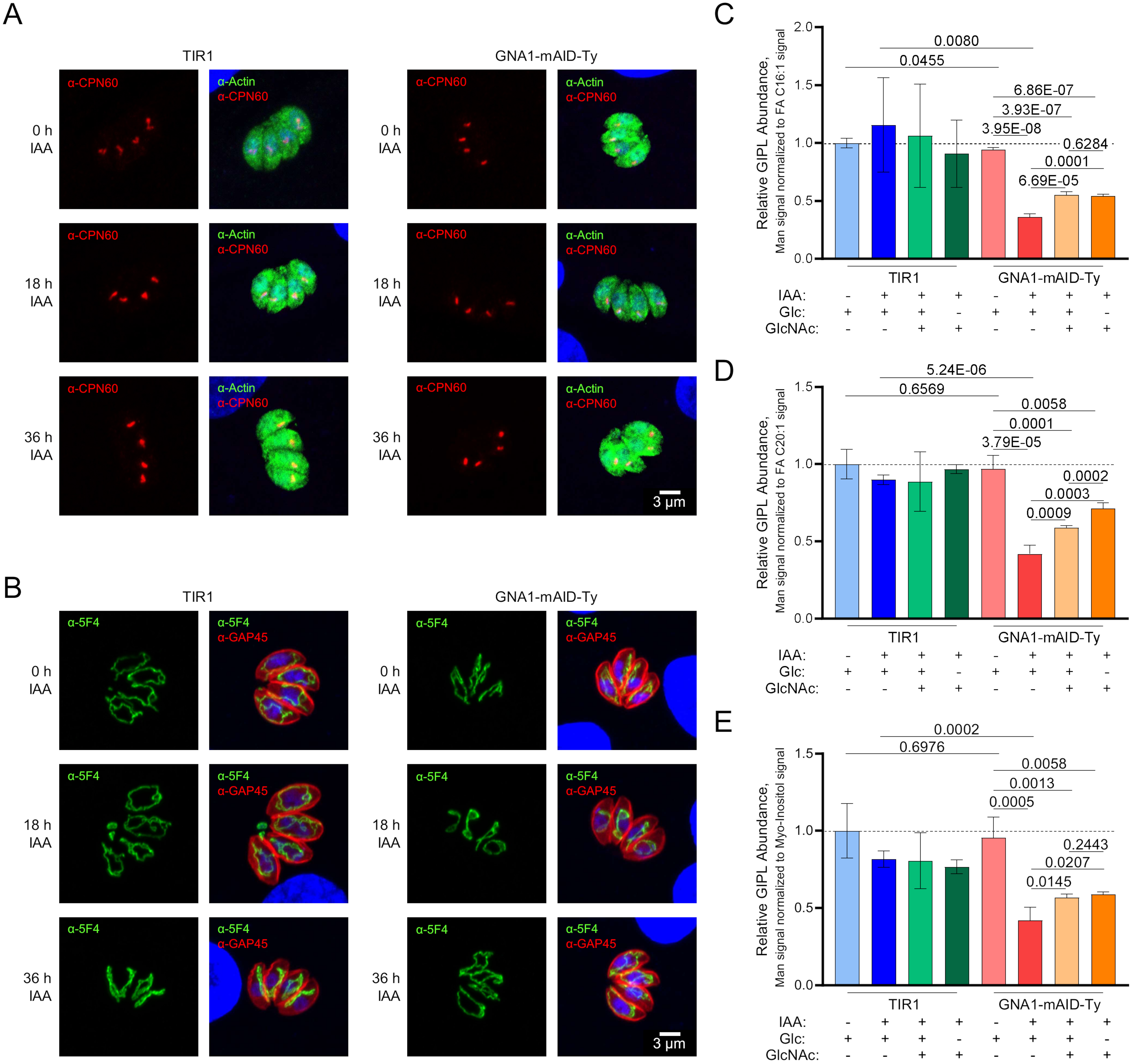

Supplement: S4 Fig — Immunofluorescence assays (IFAs) were performed after auxin (IAA) treatment for the indicated durations and after 24 hours of intracellular growth. Cells were stained with A) antibodies marking actin and the apicoplast (chaperonin 60, CPN60) or B) antibodies staining the pellicle (GAP45) and the mitochondrion (5F4). Intactness of the apicoplast and mitochondrion was determined in three technical replicates of a single experiment. Images shown are representative of these analyses. C-E) Glycoinositolphospholipid (GIPL) quantification was validated by employing a distinct extraction protocol based on butanol-partitioning and normalising the signal obtained for GIPL-derived mannose to different fatty acids (C16:1, C or C20:1, D) or myo-inositol (E). (TIF) [file ppat.1011979.s004.tif]
